# Observations upon Intra-Cranial Tuberculosis in Childhood

**Published:** 1907-08-31

**Authors:** William P. S. Branson

**Affiliations:** Assistant Physician, East London Hospital for Children


### OBSERVATIONS UPON INTRA-CRANIAL TUBERCULOSIS IN CHILDHOOD.

By WILLIAM P. S. BRANSON, M.D. M.R.C.P., Assistant Physician, East London Hospital for Children.

(*Continued from page* 551.)

I have previously reviewed the principal features of the commonest variety of intra-cranial tuberculosis, namely, miliary tuberculous meningitis. It remains to deal with two rarer forms—caseating meningitis, and caseous tumour of the brain.

Caseating Meningitis.

This lesion in its typical form is quite unusual, but presents a remarkable clinical picture which entitles it to separate consideration. It stands midway between miliary meningitis and caseous tumour, and partakes of the characters of both, for the lesion is a caseation of the meninges amounting almost to a diffuse tumour. The vertex is the region principally affected, the meninges in this neighbourhood being the seat of a greenish deposit, most in evidence along the longitudinal fissure. This deposit dips into the sulci, and involves the brain-tissue to a considerable depth. The symptoms are well demonstrated by the following account of two illustrative cases.

I. A girl of four years, two days before her admission to the East London Hospital, while walking in the street complained that her knees were giving way. She was assisted home, was able to walk again by the evening, and on the following day appeared to be quite well. On the day of her admission, however, she suddenly began to scream, complaining of pain in the left arm and leg, which were observed to twitch for a few minutes. There was no loss of consciousness. She was brought to the hospital later in the day, presenting no paresis, nor any symptom of note except a slight anæmia. The next day, while walking in the ward, she cried out and stumbled. She was found to be conscious, but the left leg was rigid. This tonic phase lasted for about thirty seconds, and was followed by a clonic spasm of about two minutes' duration, which left the left leg weak, and abolished both the knee-jerk and the plantar reflex for a time. The left arm passed through a short phase of rigidity, unattended by clonus. Within five minutes she was able to stand, and later in the day was to all appearance well, and playing with her toys. During the next few days she underwent many similar seizures, which gradually became of longer duration, began to involve the face and neck on the opposite side, and were accompanied by an increasing irritability. At this stage there was no optic neuritis. On the eighteenth day of the illness the head became retracted, and on the twenty-fifth day the breathing irregular. She died on the twenty-sixth day, having been constipated throughout, but without having vomited once. Autopsy showed a greenish tuberculous deposit in the pia-arachnoid, situated on the vertex in the neighbourhood of the right Rolandic area and extending on to the marginal convolution.

II. A girl of eight years, having been out of sorts for three weeks, was attacked one day by a sensation of "pins and needles," which affected the whole of the right side, but was not associated with any loss of power. At this time she became constipated. Ten days later an attack of clonic spasm seized the right arm and leg, leaving the former weak and the latter useless. Nine days later again a recurrence of the spasm left her completely hemiplegic. At the time of her admission to the hospital, two days after the last seizure, she presented a right hemi-paresis with exaggerated reflexes. The plantar reflex was extensor on the right side, flexor on the left. There was no ocular paralysis, nor optic neuritis. Shortly after her admission she began to vomit, grew fretful, and gradually incoherent. On the thirtieth day from the onset of the formication the left leg became spastic, and control of the sphincters was lost. On the thirty-seventh day the vomiting abated, the patient being at this time semi-conscious. On the forty-first day strabismus was noted, and she died comatose on the forty-eighth day. Post-mortem examination showed a diffuse caseous deposit situated over the left Rolandic area and following the line of the longitudinal fissure. There was, in addition, a miliary meningitis of the base of the brain.

The lesion with which we are concerned is therefore characterised clinically by a relative chronicity quite foreign to the common type of tuberculous meningitis, whose duration seldom exceeds three weeks; the lesion is mainly vertical instead of basal, and the symptoms have a Jacksonian quality not met with in the miliary variety of meningitis. The point which deserves especial stress is that in the early stages of the malady the motor phenomena are quite evanescent and give way to interludes of apparently perfect health, which may be very baffling to the diagnostician. In the absence of expert observation these Jacksonian seizures may easily be classed vaguely as "fits" and ascribed to epilepsy, with the result that the diagnosis is upset with embarrassing rapidity afterwards.

### Caseous Tumour of the Brain.

Tuberculous masses form a large majority of brain-tumours in childhood. They may be single or multiple, and consist of a dense, greenish caseous substance which seldom shows any tendency to disintegrate. These collections occur most frequently in the cerebellum or about the region of the pons Varolii, but they may be found anywhere. They may be found after death in the brains of children who have shown no signs of such a lesion during life. Of twelve instances in which post-mortem examination revealed the presence of caseous tumour, six only gave signs during life of anything more than the terminal meningitis which commonly concludes the disease. This latency is especially frequent, as might be expected, in infants of very tender age, but is in the main dictated by the size and situation of the tumour.

There are few symptoms specific to tuberculous as compared with other tumours. Like the latter, they commonly give rise to the classical symptoms, headache, vomiting, and optic neuritis, leading not infrequently to complete blindness. They may involve the motor paths and lead to corresponding paralyses or pareses. When situated in the pons Varolii they may give rise to a condition of semi-coma by which the accompanying motor disabilities are disguised, the dullness depending upon internal hydrocephalus produced by blocking of the aqueduct of Sylvius. When situated in the cerebellum they produce the ataxia peculiar to lesions of that organ. But there are many silent regions of the brain in which such tumours may exist unsuspected for long periods. This chronicity and capacity for remaining in abeyance is the most specific thing about tuberculous tumours of the brain, as may be seen from the following examples. A girl of three and a half years was attacked by a left-sided convulsion which lasted for five hours. She appeared to recover and remained free from further convulsions, though in indifferent health, for fourteen months, when she died of tuberculous meningitis. Autopsy showed caseous tumours in the left temporal and left occipital lobes. Again, a girl of five years died after a long illness, as a result of caseous tumours in the right temporal lobe and in the left inferior cerebellar peduncle. The first sign of the disease had been a sudden attack of vomiting two years previously, followed almost immediately by a convulsive seizure chiefly involving the left side. The left hand remained slightly tremulous until the occurrence of similar convulsions three months later. These attacks recurred at intervals of some months, the last some ten months before her death. Such cases as these emphasise the potential gravity of "fits" in children, especially if these occur at an age at which convulsions apart from definite epilepsy are the reverse of common.

